# A Rapid, Reliable and Reproducible Protocol for DNA Degradation in Genetic Applications

**DOI:** 10.3390/cells14211683

**Published:** 2025-10-27

**Authors:** Lena Ewers, Walther Parson

**Affiliations:** 1 Institute of Legal Medicine, Medical University of Innsbruck, Muellerstraße 44, 6020 Innsbruck, Austria; lena.ewers@i-med.ac.at; 2 Forensic Science Program, The Pennsylvania State University, University Park, PA 16802, USA

**Keywords:** degraded DNA, mitochondrial DNA, Short Tandem Repeats, human identification, real-time PCR, forensic genetics

## Abstract

Degraded DNA is frequently encountered in biological samples due to mechanisms that favor the decomposition process and thus the fragmentation of DNA due to (extreme) environmental conditions. The reduced size of the DNA fragments may hamper the performance of genetic tests. This is why developmental evaluation and validation experiments of new markers and new technologies also involve the analysis of artificially degraded DNA. This study presents a method to reproducibly generate degraded DNA in only five minutes. Different concentrations and volumes of DNA extracted from blood were irradiated by UV-C light. This led to a gradual decrease in DNA fragment size that is typically targeted in genetic applications. An increase in DNA extract volume showed only little effect on the degradation performance; the starting DNA amount slightly shifted the observed degradation pattern. The process was assessed using degradation-sensitive quantitative real-time PCR and Short Tandem Repeat analysis. Repeated experiments resulted in comparable degradation patterns that were suitable for mimicking degradation states in biological samples and for evaluating genotyping applications.

## 1. Introduction

Molecular genetic testing (e.g., DNA profiling) serves as technological basis for investigations in forensic, medical and population genetic applications. Human genetic variation is used to acquire comprehensive information about individuals, including identity, relatedness, medical conditions, biogeographic ancestry, physical appearance and many more. The quality of the investigated DNA varies. It may have suffered from environmental conditions that led to (high) fragmentation, which may pose analytical challenges. This is particularly true in biological samples retrieved from crime scenes and human remains that frequently exhibit both low DNA quality and quantity and extends to embedding and fixation of tissue samples for medical genetic purposes. The reasons for fragmentation are manifold in nature and include degradation processes that are caused by various factors, such as physical interactions (e.g., radiation), chemical reactions (e.g., hydrolysis and oxidation), enzymatic activity of nucleases, or microbial activity in general. A common result of all these processes, relevant to genetic analyses, is the damage to the structural integrity of the DNA molecule, leading to (severe) reduction in DNA fragment sizes available for genotyping [1].

Short Tandem Repeat (STR) markers are widely applied in genetic disciplines. They typically display fragment sizes between 100 to 450 bp [2]. In addition to other factors, their successful analysis primarily depends on the availability of intact target molecules of that size range. With increasing degradation of the nuclear (n)DNA, STR profiles become incomplete, resulting in loss of discriminatory power (e.g., evidential strength; [3,4]). To rescue statistically powerful DNA results, alternative nDNA markers can be tested, such as short insertion/deletion (indel) polymorphisms, e.g., [5,6] or single-nucleotide polymorphisms (SNPs; e.g., [7,8,9]). Samples that are highly degraded and do not contain enough nDNA may contain enough intact mitochondrial (mt)DNA, which carries far less genetic information but often serves as last resort for genetic tests because of its higher copy number of target molecules per cell and its increased resistance to degradation [10,11,12,13,14]. MtDNA variation is based on SNPs, which can be successfully retrieved from degraded DNA down to less than 50 bp [15,16,17].

As degradation plays an immanent role in genetic analysis of challenged samples, new molecular genetic tools and assays are commonly also tested for fragmented DNA. Naturally degraded samples represent a valid resource for this purpose; however, their degradation state cannot be well defined. This emphasizes the importance of establishing a reliable method for generating artificially degraded DNA with expectable DNA fragment sizes to mimic natural degradation so that methods can be tested and optimized more effectively.

## 2. Materials and Methods

### 2.1. Sample and Sample Preparation

DNA was extracted from whole blood donated by a volunteer at our institute under informed consent using the QIAamp DNA Blood Maxi Kit (Qiagen, Hilden, Germany) according to the manufacturer’s recommendations.

DNA was quantified via SD quants, a real-time quantitative PCR assay that targets one nuclear and two differently sized mtDNA regions (69 bp and 143 bp; [18]). DNA was diluted with low TE (10 mM Tris, 0.1 mM EDTA, pH 8) to prepare sample stock solutions of the following concentrations: 1 ng/µL, 7 ng/µL and 14 ng/µL. Aliquots of 10 µL or 20 µL were prepared using 0.6 mL microtubes (Axygen, Corning Life Sciences, Corning, NY, USA).

### 2.2. Experimental Set-Up

A custom-made UV-C irradiation unit (Figure S1; Unilab Technologies GmbH, Innsbruck, Austria) equipped with three 30 W G13 germicidal lamps in the UV-C spectrum with the main spectral line at 254 nm (PURITEC HNS UV-C; OSRAM GmbH, Munich, Germany) was used to expose the samples to UV-C light at a photometric power of 12 W. It was designed with an outer cover made of polished metal that completely shields the interior from other external influences, with dimensions of 30.8 cm × 100.6 cm. Considering health and safety, the UV-C irradiation unit was placed under a laboratory hood with a protective screen for all experiments. Sample aliquots were positioned in microtubes laid on their side on the laboratory bench under the UV-C light source at a distance of ~11 cm from the lamps. Exposure time was monitored manually using a stopwatch and stopped at 30 s time intervals, i.e., degradation stages, when the lamps were switched off and aliquots were removed. Ten 10 µL aliquots [7 ng/µL] of the DNA extract were irradiated in two technical replicates (N = 10, *n* = 20) over a time period of 5.0 min. During this degradation interval, two replicates were removed every 30 s. All aliquots were irradiated simultaneously, whereby replicates of the same degradation step were placed next to each other under the UV-C light source to ensure that conditions were as reproducible as possible.

In order to test the influence of sample volume and DNA concentration and for evaluating reliability and reproducibility, three experiments were conducted with the physically same DNA extract: I. 20 µL aliquots [7 ng/µL] (N = 10, *n* = 10), 10 µL aliquots [7 ng/µL] (N = 10, *n* = 20) and 10 µL aliquots [14 ng/µL] (N = 10, *n* = 20), II. 10 µL aliquots [1 ng/µL] (N = 10, *n* = 20), III. 10 µL aliquots [1 ng/µL] (N = 10, *n* = 20) after ~1.5 years of storage. For all three experiments the same procedure was carried out as described above.

Degraded DNA aliquots were quantified using SD quants on a QuantStudio 5 Real-Time PCR System (Thermo Fisher Scientific (TFS), Waltham, MA, USA) and analyzed by STR typing (AmpFLSTR NGM SElect PCR Amplification Kit; TFS) on an ABI 3500xL Genetic Analyzer HID (Applied Biosystems by TFS). DNA quantitation and STR profiling were carried out immediately after the degradation processes. The experiments were performed at room temperature, and for short-term storage, aliquots were kept dark and at 4 °C.

### 2.3. Analysis

The loss of mtDNA copies, i.e., mtDNA Genome equivalents (mtGE), as a function of the exposure time to UV-C radiation was determined by quantification via SD quants. As this quantitative PCR included two differently sized mtDNA targets, a degradation index (DI) was calculated by dividing the DNA amount of the long target (mt143bp) by that of the short target size (mt69bp). Calculations and plotting were done with either Microsoft Excel or R-Studio (v4.3.0). Capillary electrophoretic results were analyzed using the GeneMapper™ ID-X Client software v1.6 (TFS).

## 3. Results and Discussion

Degraded DNA is commonly encountered in genetic analyses. It is crucial to understand the limitation of genetic testing when (highly) fragmented DNA is subject to investigations. This is why evaluation and validation experiments on (new) marker panels and assays should extend to the examination of degraded DNA. This kind of DNA is found in old, decomposed or chemically treated samples, but the quantity and quality of the DNA available in such specimens varies and is difficult to standardize. Therefore, artificially degraded DNA can be helpful when generated in a reproducible way.

We have examined alternative methods to produce artificially degraded DNA, including sonication and Turbo DNase treatment [19,20]. Sonication did not result in any considerable DNA degradation, even after 8.0 h treatment, whereas enzymatic digestion with 0.1 U Turbo DNase resulted in complete DNA digestion within only 0.5 min. However, neither method led to reproducible degradation patterns when repeated after a longer time period (e.g., after some months). Consistent with these observations, the evaluation of STR profiles revealed no gradual decrease in amplification success or allelic signal quality that would indicate a progressive DNA degradation over time. Given the strong absorption of DNA in the UV-C spectrum, leading to photochemical changes in the nucleic acid level [21], UV-C light at 254 nm was explored as a more reproducible method for generating artificially degraded DNA. The most important UV-C-induced DNA alterations are cyclobutane pyrimidine dimers between neighboring pyrimidines and 6-4-photoproducts, which result from covalent linkage of pyrimidines [21,22,23]. Oxidative lesions (e.g., 8-oxodGuo) and strand break formation occur at lower rate at this wavelength and are thought to arise indirectly [21,24,25,26]. These lesions to the DNA molecule reduce the amount of intact, amplifiable DNA available for PCR-based genetic analysis.

**Figure 1:**
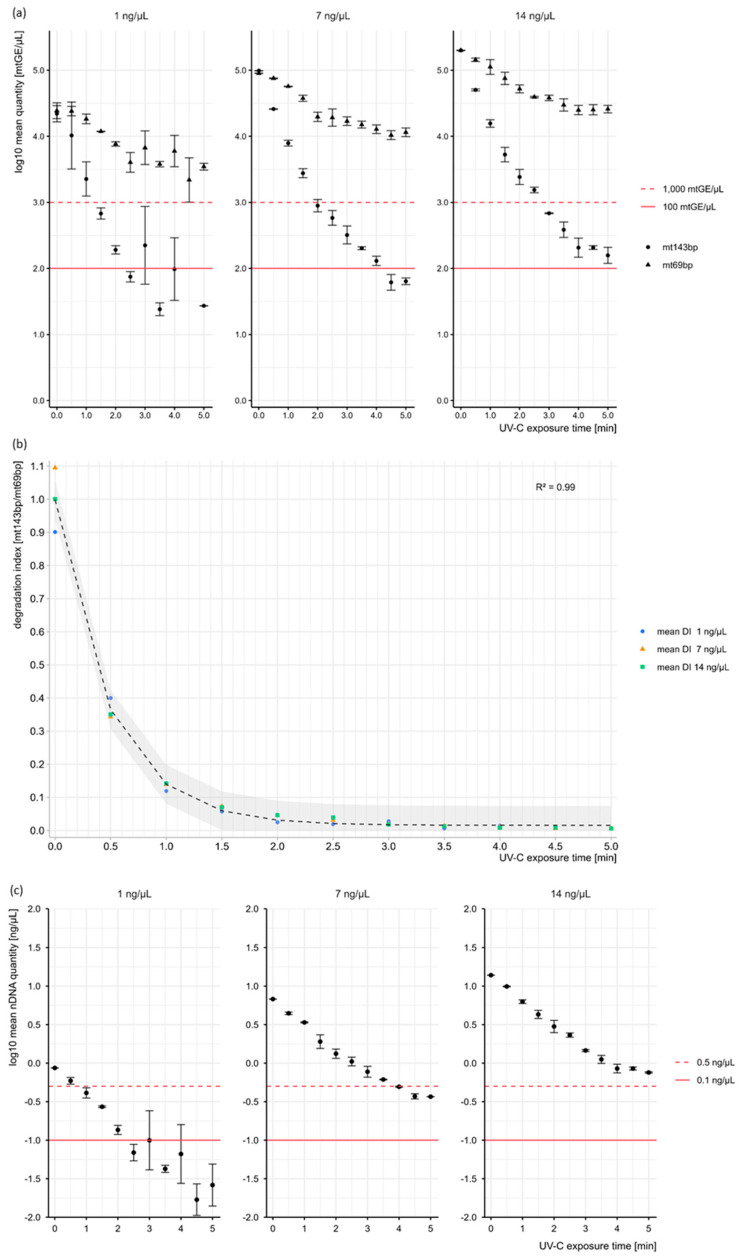
(**a**) Size-dependent mtDNA copy number loss under UV-C exposure. The plots visualize the increasing fragmentation of DNA with progressive degradation based on the remaining quantities determined after 30 s-time intervals. The dashed and solid lines mark the 1000 and 100 mtDNA copy thresholds. Error bars represent the respective standard deviation (SD). (**b**) Temporal dynamic of mtDNA degradation indices under UV-C exposure. Mean degradation indices (DI) for the different DNA concentrations fitted with an exponential regression model (pseudo R^2^ = 0.99). Predicted DI values are shown as dashed line. Almost all data points lie within the 95% confidence interval. (**c**) Quantity loss of the nDNA target under UV-C exposure. The log_10_ mean quantities of the nDNA target (nuRNU) are shown across different UV-C exposure times. The range between 0.5 ng/µL and 0.1 ng/µL, corresponding to partial STR profile recovery, is highlighted. Error bars represent the respective standard deviation (SD).

A gradual decrease in DNA quantity with UV-C exposure time was observed for all samples (Table 1 and Table 2; Figure 1a–c; Tables S1a,b, S2a,b and S3a; Figures S2–S6). This decrease was largely independent of the DNA starting amount tested here and led to similar relative losses for the three different DNA concentrations with the respective nDNA and mtDNA target sizes. ANOVA testing confirmed a highly significant dependence of relative quantity loss on UV-C exposure time, while no significant differences were found between the series, supporting the conclusion that the investigated series exhibited similar degradation patterns.

Technical replicates at 0.5 min of UV-C exposure in the 1 ng series (mtDNA targets) slightly deviated from this trend. These deviations are likely attributable to natural variation in the initial DNA quantities among aliquots, arising from both minor discrepancies during sample preparation and the inherent variability in the small amounts of DNA used in the qPCR, which could also contribute to stochastic effects (mt69bp: 29,054 and 19,753 mtGE/µL; mt143bp: 26,329 and 17,651 mtGE/µL). Consequently, the mean quantity was determined to be 10,303 ± 9876 mtGE/µL (RSD 95.8%), with a mean relative loss of only 53.15% for the mt143bp target (7 ng series: 73.75%; 14 ng series: 74.78%) at 0.5 min of UV-C exposure (Table 1 and Table 2; Tables S1a,b and S2a,b). In general, the relative loss of mtDNA at 69bp after 5 min of UV-C exposure was consistently lower than that observed for the mt143bp target, with ~90% of the relative quantity loss occurring within 1 min of irradiation (Table 2; Tables S1b and S2b).

Table 1 displays the UV-C exposure-dependent decline in the mean concentrations of the mtDNA targets (mt143bp and mt69bp) and the nuclear target (nuRNU) in the 7 ng series, along with their respective standard deviations. The quantification data of the mt143bp target show a rapid decrease in mtDNA amount within the first 2 min at which a critical value (1000 mtDNA copies) for downstream mtDNA typing is reached (Figure 1a). Successful PCR-based massively parallel sequencing applications typically require between 3000 and 5000 mtDNA copies based on the mt143bp target (TFS, Precision ID Panels with Ion S5TM System APPLICATION GUIDE—Publication number MAN0017770; [27]). The decline of the mt69bp target was less pronounced, and a value of 10,000 mtDNA copies remained detectable even after UV-C treatment for 5 min (Table 1; Figure 1a), which is also an important observation when developing DNA-free laboratory procedures for genetic applications. With increasing UV-C exposure time DI values progressively decreased, indicating a significantly lower amount of intact long fragments compared to shorter fragments. This emphasizes the susceptibility of longer DNA targets to degradation.

The 1 ng series exhibited greater standard deviations and dropouts (both replicates at 4 min and one replicate at 5 min), reflecting the increased variability and susceptibility of low-concentration samples to stochastic effects (Figure 1a; Table S1a,b). Beginning at 3 min of UV-C exposure, fluctuations in the measured quantities were observed, while the other series exhibited a continuous decrease in DNA quantity with lower standard deviations. All tested concentrations exhibited the same gradual degradation pattern with a slight temporal shift in the point at which the 1000 mtGE/µL threshold for the mt143bp target was reached. This can be attributed to the varying initial amounts of target molecules.

Since the mtDNA degradation index exhibited a consistent temporal decline under UV-C exposure across all tested concentrations, an exponential regression model was applied to the entire dataset (Figure 1b). To account for the observed stagnation of degradation upon reaching the biological plateau at DI = 0, a decay limit was incorporated. The exponential regression model demonstrated a good fit to the data, as indicated by a high pseudo-R^2^ value (0.99) and low AIC (127.42), BIC (−121.68) and RSE (0.03). Most values fell within the 95% confidence interval, further supporting the model’s reliability. Based on this, the time point of UV-C exposure at which the mt143bp target reached the 1000 mtGE/µL threshold was estimated, ensuring reliable extrapolation within the observed data range. Extrapolated time estimates for the 7 ng series (1.89 min) and 14 ng series (2.81 min) showed good agreement with the quantification data, which indicated time points between 1.5 to 2.0 min and 2.5 to 3.0 min, respectively. For the 1 ng series, the estimated time point of 1.63 min was slightly higher than the quantifications suggested. However, this discrepancy can be attributed to the increased sensitivity to stochastic effects at lower concentrations.

To provide a complete view of nucleic acid degradation, Figure 1c illustrates the behavior of the nDNA target (nuRNU, 70 bp) under the same UV-C exposure conditions, although our analyses primarily focused on mtDNA. The degradation trend closely parallels that of the mt69bp target but occurs at lower absolute levels, reflecting the lower copy number of nuclear DNA. This is consistent with Table 2, which demonstrates a similar relative quantity loss for nuRNU and mt69bp. The apparently stronger initial decrease in the nuRNU target can be explained by its reduced abundance, which renders the same UV-C dose comparatively more effective.

As part of the evaluation of the reproducibility and reliability of this method, we not only examined different DNA concentrations (Figure S2) but also different volumes of DNA extract (Figure S3). The results indicated no visible effect of DNA volume on the degradation process; however, only two volumes (10 µL and 20 µL) were tested, with the 20 µL volume, analyzed in a single replicate (Figure S3). UV-C radiation of DNA extracted from saliva exhibited similar degradation patterns, confirming applicability to other primary DNA sources (Figure S4). As saliva DNA was quantified from pooled samples without biological replicates, standard deviations were omitted since they would reflect only technical variation. These results demonstrate that the UV-C degradation protocol can be applied to other primary DNA sources, while tissue samples were excluded, as extraction could compromise the reproducible generation of DNA at defined degradation stages, which is essential for testing and optimizing downstream analyses.

To further evaluate the reproducibility and reliability of the degradation method, a storage stability test was conducted. The mtDNA and nDNA quantities from the 1 ng series were measured after ~1.5 years of storage to assess potential long-term effects on degradation patterns. The analysis revealed that prolonged UV-C exposure increased the disparity in mtDNA quantities, leading to a more pronounced loss. In contrast, nDNA loss was observed only within the first 1.5 min of UV-C exposure; while beyond 2 min, the quantities remained stable (Table S3, Figure S5).

STR-electropherograms showed typical degradation patterns (“ski-slope effect”) with an increasing extent over exposure time (Figure S6a–d). We observe the typical degradation phenomenon, where the signal intensity (i.e., the peak heights) decreases with larger fragment sizes up to complete marker dropouts.

The effect of UV-C-induced damage on DNA with respect to forensic analysis has previously been investigated by Hall and Ballantyne [28] and Rahi et al. [29].

In the study conducted by Rahi et al. [29], 10 µL blood stains, dried for 24 h on FTA cards, were placed in 2 mL tubes held in a rack and exposed to a UV-C (254 nm) at a distance of 3.2 cm with the tube caps open. Exposure lasted up to 2 h in 20 min intervals. DNA extracts were then quantified with the Quantifiler Duo Quantification Kit (TFS), and STR amplification was performed using the AmpFLSTR Identifiler PCR Amplification Kit (TFS). The kit includes 15 STR markers and the Amelogenin sex determination marker. Amplification was carried out using 1 ng of DNA input, as determined by the prior quantification. Amplification products were separated using an ABI PRISM Genetic Analyzer and analyzed with the GeneMapper ID software (v3.2.1). STR analysis revealed that even after maximal UV-C exposure time of 2 h, complete profile loss did not occur. However, high-molecular-weight markers were completely lost after 20 min of UV-C exposure, while medium-molecular-weight markers began to degrade at 20 min and were fully degraded by 60 min.

Besides overnight-dried 50 µL blood stains on Whatman No. 3 cards, Hall and Ballantyne [28] also exposed 100 ng/µL naked DNA and dehydrated naked DNA in solution (20 µL aliquots) to UV-C light. Samples were inserted into 1.5 mL microcentrifuge tubes, laid horizontally with the caps closed and positioned at the bottom of a UVXL-100 Crosslinker, which emits a sharp peak of irradiance at 254 nm. Exposure times ranged from 30 s to 102 h. DNA from blood stains was extracted, and both sample types were analyzed by gel electrophoresis. Genotype profiles were obtained by amplification using the AmpFLSTR PCR Amplification Kit (TFS), which includes 9 STR markers and Amelogenin. Amplification products were separated on an ABI PRISM 3100 CE (Applied Biosystems by TFS) and analyzed with the GeneScan software (v3.1.2). After 102 h of UV-C exposure, blood stain samples exhibited a complete profile loss. A partial profile was still detectable after 12 h, whereas samples exposed for 8 h retained a profile of good quality. Among the five STR loci that dropped out after 8 h of UV-C exposure were FGA and CSF1PO (both high-molecular-weight), as well as D13S317 and D7S820 (both medium molecular weight). Notably, the same markers showed drop-out after just 1 h of exposure in the study conducted by Rahi et al. [29]. Possible reasons for the differences observed in the degradation sensitivity of blood stains between the two studies could include variations in the volume of blood used for the stain preparation, the orientation of the tubes and the status of the tube caps during UV-C exposure as well as the differences in UV-C intensity. The UV-C intensity was calculated to be 1.7 mW/cm^2^ in the study by Hall and Ballantyne [28] and 1.2 mW/cm^2^ in the study by Rahi et al. [29], representing a 40% deviation.

For the naked DNA, Hall and Ballantyne observed the first locus drop-out (D7S820) and reduced signal intensity after 2 min of UV-C exposure, with complete profile loss after 16 min at a UV-C dose of 1.664 J/cm^2^.

In the present study, reduced signal intensities as well as allele and locus drop-outs in DNA aliquots were already detected at shorter UV-C exposure times. This could be explained by the substantially lower DNA concentrations (100-, 14- and 7-fold reductions) and the smaller volume (10 µL) but also by the markedly higher UV-C intensity (6.7-fold) resulting in a UV-C dose of 1.743 J/cm^2^ being reached after only 2.5 min of UV-C exposure.

Generally, naked DNA seems more prone to photochemical reactions than DNA in blood stains, which is explained by the structural integrity of this sample type [28,29].

External factors such as temperature, humidity, or ionic strength can in principle influence DNA structure and UV-C–induced photoreactivity [25,28,30]. In our study, all experiments were performed on isolated DNA in low-TE solution, in closed tubes, under consistent laboratory conditions. Repeated experiments on different days showed highly reproducible degradation patterns (assessed via STR profiles), suggesting that minor variations in environmental factors do not substantially affect the outcome.

## 4. Conclusions

In summary, our results demonstrate that UV-C exposure under our methodological setup produces artificially degraded DNA in a rapid, reliable, and reproducible manner. This technique is ideal for studies using artificially degraded DNA, as the fragmentation patterns occur within just a few minutes. Moreover, the resulting DNA fragments fall within the size range observed in naturally degraded DNA relevant to PCR-based genotyping applications, making this approach a valuable model for evaluation and validation purposes.

## Figures and Tables

**Table 1:** Decline in apparent DNA quantity with UV-C exposure time. Mean loss of mt143bp and mt69bp targeted mtDNA and 70 bp nDNA (nuRNU) of the 7 ng series.

UV-C Exposure [Min]	mt143bp [mtGE/µL]			mt69bp [mtGE/µL]			nuRNU [ng/µL]		
	Mean	Sd		Mean	Sd		Mean	Sd	
0	98,556	- ^a^		89,995	- ^a^		6.79	- ^a^	
0.5	25,875	±	210	75,524	±	1016	4.44	±	0.17
1	7880	±	802	56,881	±	371	3.38	±	0.06
1.5	2763	±	432	37,587	±	4067	1.92	±	0.39
2	892	±	191	19,735	±	3155	1.33	±	0.18
2.5	581	±	148	19,167	±	5692	1.05	±	0.14
3	320	±	99	16,978	±	2543	0.78	±	0.13
3.5	203	±	10	15,032	±	1840	0.61	±	0.01
4	130	±	21	12,786	±	1914	0.49	±	0.01
4.5	62	±	17	10,413	±	1614	0.37	±	0.03
5	64	±	7	11,521	±	1730	0.37	±	0

^a^ Single replicate.

**Table 2:** Mean percentage loss relative to the initial quantity over UV-C exposure time. Mean loss of mt143bp and mt69bp targeted mtDNA and the nDNA (nuRNU) of the 7 ng series.

UV-C Exposure [min]	Quantity Loss [%]		
	mt143bp	mt69bp	nuRNU
0.5	73.75	16.08	34.64
1	92.00	36.80	50.22
1.5	97.20	58.23	71.70
2	99.10	78.07	80.39
2.5	99.41	78.70	84.47
3	99.68	81.13	88.54
3.5	99.79	83.30	90.97
4	99.87	85.79	92.73
4.5	99.94	88.43	94.53
5.0	99.94	87.20	94.59

## Data Availability

The data presented in this study are available on request from the corresponding author. The data are not publicly available due to privacy restrictions.

## Supplementary Materials

The following supporting information can be downloaded at: https://www.mdpi.com/article/10.3390/cells14211683/s1, Figure S1: Schematic documentation of experimental workflow; Table S1a: Decline in DNA quantity with UV-C exposure time; Table S1b: Mean percentage loss relative to the initial quantity over UV-C exposure time; Table S2a: Decline in DNA quantity with UV-C exposure time; Table S2b: Mean percentage loss relative to the initial quantity over UV-C exposure time; Figure S2: Comparison of the degradation pattern of differently concentrated samples; Figure S3: The influence of sample volume on the degradation effect; Table S3: Comparison of mtDNA quantities in the 1 ng series after 1.5 years of storage at −20 °C; Figure S4: UV-C–induced DNA degradation in saliva DNA extracts; Figure S5: Storage test; Figure S6a–d: STR-Profile.
